# Photo-responsive nanozymes: from photocatalytic mechanisms to precision therapy

**DOI:** 10.3389/fchem.2026.1878757

**Published:** 2026-06-16

**Authors:** Ting Linghu, Xubin Gu, Guochao Chang, Xiang Sun, Ruiping Zhang

**Affiliations:** 1 The Radiology Department of Shanxi Provincial People’s Hospital Affiliated to Shanxi Medical University, Taiyuan, Shanxi, China; 2 Institute of Medical Technology, Shanxi Medical University, Taiyuan, Shanxi, China; 3 Modern Research Center for Traditional Chinese Medicine, Shanxi University, Taiyuan, Shanxi, China; 4 School of Basic Medical Sciences, Shanxi Medical University, Taiyuan, China

**Keywords:** catalytic enhancement strategy, catalytic mechanism, photocatalysis, photo-responsive nanozymes, precision therapy

## Abstract

Conventional nanozymes still suffer from limited catalytic efficiency and poor substrate selectivity, which impede their clinical translation. Recently, photo-responsive nanozymes have emerged as a promising platform for precision biomedicine by integrating photocatalytic processes with enzyme-mimetic catalysis, enabling spatiotemporally controlled enhancement of catalytic activity. In this review, we highlight strategies for incorporating emerging photocatalytic technologies into the rational design of nanozymes and systematically elucidate the core mechanisms by which photogenerated charge carriers and plasmonic resonance facilitate nanozyme catalytic reactions. We further summarize the biomedical applications of photo-responsive nanozymes in tumor therapy, microbial eradication, inflammation regulation, and biosensing. Finally, we analyze current challenges and future perspectives regarding biocompatibility, catalytic efficiency, and deep-tissue light penetration, thereby providing a theoretical foundation for the development and clinical translation of high-performance photo-responsive nanozymes.

## 1 Introduction

Nanozymes, defined as nanomaterials with enzyme-mimetic catalytic activities, have attracted increasing attention in biomedical applications, including tumor therapy, microbial eradication, anti-inflammatory therapy, and biosensing, owing to their high stability, low production cost, and suitability for scalable fabrication, storage, and transportation (Xie P. et al., 2022; Zhang et al., 2021; Zhang H. et al., 2025). However, compared with natural enzymes, conventional nanozymes still face major limitations in activity tunability, substrate selectivity, and biocompatibility (Wang Q. et al., 2021). In complex physiological microenvironments, their catalytic performance is readily perturbed by pH, ionic strength, and endogenous biomolecules, often resulting in catalytic efficiencies markedly lower than those of natural enzymes and unpredictable therapeutic outcomes, thereby severely limiting their application in precision medicine (Dong et al., 2023b; Feng et al., 2024; Zhang et al., 2024).

Recent advances in photocatalytic technologies have provided new opportunities for the dynamic, remote, and precise regulation of nanozyme activity (Liu X. et al., 2020; Men et al., 2026; Yang et al., 2024). Photocatalysis uses light at specific wavelengths to activate photophysical and photochemical processes in nanomaterials, thereby converting light energy into chemical energy, thermal energy, or photogenerated charge carriers. These processes can enhance or modulate nanozyme-mediated catalysis (Lin et al., 2022). Compared with thermal and chemical stimuli, light-mediated regulation offers distinct advantages, including non-invasiveness, high spatiotemporal resolution, and remote controllability. These features enable precise control over the initiation and termination of catalytic reactions from spatial and temporal dimensions. By integrating photocatalytic materials with nanozymes, intelligent responsive systems can be constructed to trigger cascade catalytic reactions under specific light irradiation. Such systems hold great promise for efficient tumor therapy, microbial eradication, and ultrasensitive biosensing (Fan et al., 2024; Liu M. et al., 2020; Ma et al., 2025; Xiao Z. et al., 2025). For example, light irradiation can reversibly switch nanozyme activity between peroxidase (POD)-like and catalase (CAT)-like modes, thereby enabling both reactive oxygen species (ROS) generation and ROS scavenging, which are essential for precision therapy (Leng et al., 2025). Furthermore, photocatalytic processes can induce multiple physicochemical effects that synergize with the intrinsic catalytic pathways of nanozymes, thereby substantially accelerating overall reaction kinetics (Shen et al., 2024; Tang et al., 2026; Wang S. et al., 2025; Wang Z. et al., 2026; Zhang et al., 2025d). For instance, photogenerated charge carriers can directly participate in redox reactions, photothermal effects can increase the local temperatures and accelerate reaction kinetics, and light-induced ROS generation can amplify nanozyme-mediated catalytic processes (Xiao F. et al., 2025). Thus, photocatalysis provides an external-energy-driven strategy for precisely regulating nanozyme activity while constructing more efficient catalytic pathways, offering a feasible route to overcome the insufficient activity and limited responsiveness of conventional nanozymes.

**FIGURE 1:**
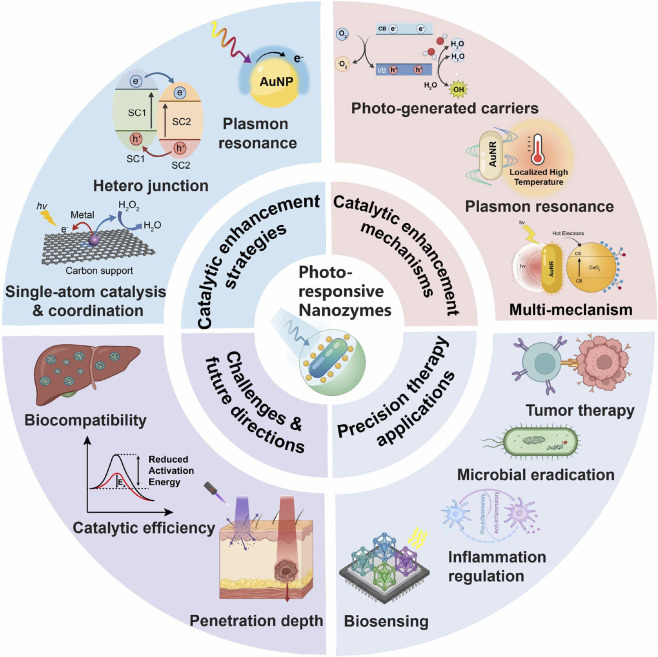
Overview of photo-responsive nanozymes. Catalytic enhancement strategies, catalytic enhancement mechanisms, precision therapy applications, challenges, and future directions.

Photocatalytic technology not only compensates for the activity limitations of conventional nanozymes but also introduces new functional dimensions. Current research in this field is gradually shifting from static catalysis toward intelligent responsiveness, highlighting the considerable potential of photoresponsive nanozymes in precision biomedicine (Figure 1; Table 1) (Feng et al., 2025; Liu W. et al., 2025; Pan M. M. et al., 2026; Wang Z. et al., 2025; Wu et al., 2025b). This review concentrates on recent advances in emerging photocatalytic technologies and their integration into the rational design of nanozymes. We systematically elucidate the core mechanisms through which photogenerated charge carriers and plasmon resonance promote nanozyme-mediated catalytic reactions, and provide a comprehensive overview of rational design strategies, catalytic enhancement mechanisms, and biomedical applications. Finally, we discuss key challenges and future perspectives in the field of photo-responsive nanozymes, aiming to provide a theoretical basis for the development and clinical translation of high-performance photo-responsive nanozymes.

**TABLE 1:** Summary of representative photo-responsive nanozymes.

Nanozyme name	Type of nanozyme	Treatment modalities	Absorption [nm]	Catalytic mechanisms	Application	Refs
AuNR@CeO_2_	POD	PTT/CT	808	SPR/Electron transfer/Photothermal effect	Microbial eradication	Liu et al. (2021)
Bi-PCN-222 MOF	POD/OXD	CT	—	Electron transfer	Microbial eradication	Wu et al. (2023)
BPF	POD/CAT	UPCT/PDT/CDT	660	Band Engineering/Electron-hole recombination	Tumor Therapy	Qiu et al. (2026)
Fe-N-C	SOD/CAT/GSH-Px	CT	—	Electron transfer	Inflammation regulation	Ying et al. (2026)
Cu/Zn-C_6_N_6_	SOD/POD	—	Light	Electron transfer	Biosensing	Hong et al. (2026)
AuPt-FeNC@HA	GOx/CAT/POD/GSHox	CDT	—	SPR	Tumor Therapy	Guo et al. (2025)
Fe-TCPP-MOFs	GSHox	PDT/CDT	660	Band Engineering	Tumor Therapy	Fan et al. (2024)
Au_2_Pt-PEG-Ce6	CAT/POD	CDT/PTT/PDT	650/808	Electron transfer/Photothermal effect	Tumor Therapy	Wang et al. (2020)
MET-CMS@FeTA	CAT/POD/GPx	PTT	1064	Electron transfer/Photothermal effect	Tumor Therapy	Zhang et al. (2025a)
Cu_2_O@Au	GOx/POD/GPx	PTT/CDT	NIR Laser	LSPR/Electron transfer	Tumor Therapy	Li et al. (2024c)
MXene/HEO@PVP	—	PTT/PDT	NIR Laser	Band Engineering/Electron transfer	Microbial eradication	Zhang et al. (2025c)
Cu-MOG MN	SOD/POD/GPx	CDT	—	Electron transfer	Microbial eradication	He et al. (2026)
HZCB	POD	PTT/CDT	808	Photothermal effect/Electron transfer	Microbial eradication	Dong et al. (2024)
mCu-SAE@BNN6@PEG-Van	POD/OXD	PTT/CDT	1064	Photothermal effect/Electron transfer	Microbial eradication	Chen et al. (2025a)
CeO_2_@PDA-Mg@PTHrP-2	SOD/CAT	PTT	808	Photothermal effect/Electron transfer	Inflammation regulation	Shi G. et al. (2025)
Cu/Mn NC	CAT/OXD/POD	PTT/CDT	1064	Electron transfer/Band Engineering	Tumor Therapy	Li et al. (2025c)
PEG/Ce-Bi@DMSN	POD/CAT	PTT/CDT	NIR-II	Photothermal effect/Electron transfer	Tumor Therapy	Dong et al. (2020)

## 2 Photocatalytic engineering strategies for enhanced nanozyme catalysis

### 2.1 Design of plasmonic photocatalytic nanozymes

**FIGURE 2:**
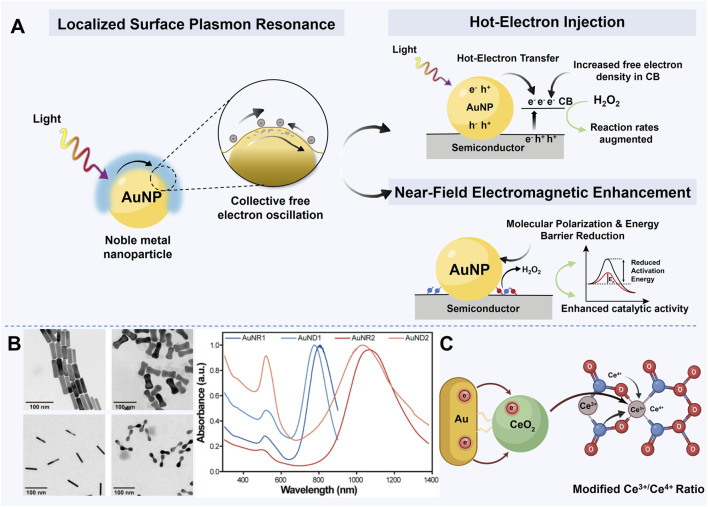
Catalytic mechanism of plasmonic photocatalytic nanozymes. **(A)** Schematic diagram of plasmonic photocatalytic nanozymes. **(B)** Representative TEM images and UV-vis-NIR absorption spectra of AuNR1, AuND1, AuNR2 and AuND2. Reproduced from (Zeng et al., 2025). Copyright 2025 Small. **(C)** The schematic diagram of the Au@CeO_2_ nanozyme photocatalytic mechanism.

Plasmon-mediated photocatalysis, particularly localized surface plasmon resonance (LSPR), has emerged as an effective strategy for enhancing the catalytic activity of photo-responsive nanozymes (Fan et al., 2023; Li et al., 2026h; Shao et al., 2025; Wu et al., 2026a; Xiao and Diao, 2025). This strategy exploits the LSPR effect generated in noble-metal nanoparticles (e.g., Au, Ag) under visible to NIR irradiation. When the frequency of incident photons aligns with the collective oscillation frequency of conduction electrons in noble-metal nanostructures, intense localized electromagnetic fields and plasmon-derived hot carriers are generated, thereby promoting interfacial charge transfer and enhancing the catalytic activity of adjacent semiconductor or carbon-based catalytic domains (Sang et al., 2024). Specifically, hot electrons can be injected from the metal component into the conduction band of the semiconductor, increasing the density of free electrons available for catalytic reactions (Li et al., 2026g). Meanwhile, plasmon-induced electromagnetic field enhancement can polarize adsorbed molecules on the semiconductor surface, lower the energy barrier of redox reactions, and accelerate reaction kinetics (Figure 2A) (Li et al., 2026d). Furthermore, plasmonic resonance can improve the long-wavelength light-harvesting capability of semiconductors through radiative energy transfer or near-field electromagnetic coupling, thereby extending their photo-response range (Yan Y. et al., 2025).

The plasmonic properties of nanozymes can be rationally regulated by precisely tailoring the morphology, size, composition, and spatial distance between noble-metal nanostructures and catalytically active sites. Such structural optimization enables fine control over the light absorption peak and enhances photothermal-photocatalytic synergy. Anisotropic nanostructures, including nanorods and nanostars, are particularly attractive because their plasmonic resonance peaks can be tuned across a broad spectral range, thereby improving light utilization efficiency (Zhou et al., 2026). For example, the absorption peak of gold nanorods (AuNRs) undergoes a red shift with increasing aspect ratio, which significantly facilitates deeper tissue penetration for efficient photothermal ablation of cancer cells and *in vivo* tumor therapy (Figure 2B) (Zeng et al., 2025). Additionally, dumbbell-like AuNR@CeO_2_ hybrid nanozymes demonstrate strong SPR responses in the NIR region. Hot-electron transfer between Au and CeO_2_ modifies the Ce^3+^/Ce^4+^ ratio, thereby significantly enhancing their peroxidase-like activity (Figure 2C) (Liu et al., 2021). Beyond conventional noble metals, semimetal bismuth also exhibits notable plasmonic properties and has been frequently employed to modify semiconductor surfaces for enhanced photocatalytic activity. This not only extends the photoresponse range to the visible region but also serves as an electron reservoir and transfer mediator, effectively suppressing the recombination of photogenerated electron-hole pairs and improving photocatalytic performance (Wu et al., 2023). Therefore, the rational design of noble-metal or semimetal nanostructures to modulate plasmonic resonance represents a powerful strategy for enhancing the photocatalytic performance of photo-responsive nanozymes.

### 2.2 Design of heterojunction photocatalytic nanozymes

**FIGURE 3:**
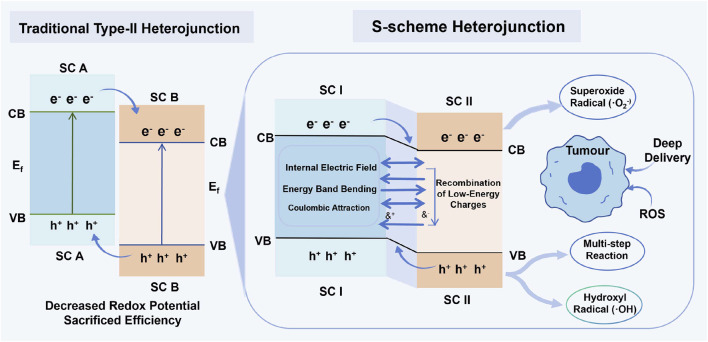
Catalytic mechanism of heterojunction photocatalytic nanozymes.

Constructing heterojunctions with spatially separated redox sites is a key strategy to achieve optimal photocatalytic performance (Dong et al., 2023a; Liu et al., 2024; Xu et al., 2025; Yang et al., 2026; Yi et al., 2025). Conventional heterojunction systems, such as Type-II heterojunctions, can promote spatial charge separation. However, they often weaken the redox potentials of photogenerated electrons and holes, which restricts their catalytic efficiency and biomedical applicability (Sayed et al., 2026). Recently, S-scheme heterojunctions have attracted increasing attention in the field of photo-responsive nanozymes due to their distinctive charge transfer pathway and superior redox-preserving capability (Zhang Y. et al., 2026). Driven by the synergistic effects of an internal electric field, interfacial band bending, and Coulombic attraction, S-scheme systems promote the recombination of low-energy, less-reactive charge carriers while retaining electrons with strong reduction capability and holes with strong oxidation capability for subsequent catalytic reactions (Figure 3) (Wang L. et al., 2023).

In biomedical applications, the introduction of S-scheme heterojunctions has substantially expanded the potential of photo-responsive nanozymes for efficient and selective catalytic therapy. For example, integrating S-scheme heterojunctions with Fe-based nanozyme catalysis in a photo/sono/magneto-responsive ternary hetero-nanocrystal platform (BPF) enables deep tumor delivery, sustained ROS amplification, and multimodal imaging-guided therapy, which provides an advanced strategy for the precise treatment of solid tumors (Qiu et al., 2026). Similarly, CeO_2_/FCN composites with S-scheme heterojunctions can couple photocatalytic processes with enzyme-mimetic reactions, thereby increasing the concentration of reactive species and enhancing photo-enzyme synergistic catalysis (Feng et al., 2023). Moreover, the spatially separated strong oxidative and reductive sites in S-scheme heterojunctions can be respectively utilized for ROS generation and substrate activation, enabling the efficient coupling of multi-step cascade catalytic reactions (Miao et al., 2026). Therefore, S-scheme heterojunctions fundamentally resolve the trade-off between charge separation and redox capability in conventional heterojunctions, providing a theoretical basis for the design of highly efficient photo-responsive nanozymes.

### 2.3 Single-atom catalysis design and coordination engineering strategy

**FIGURE 4:**
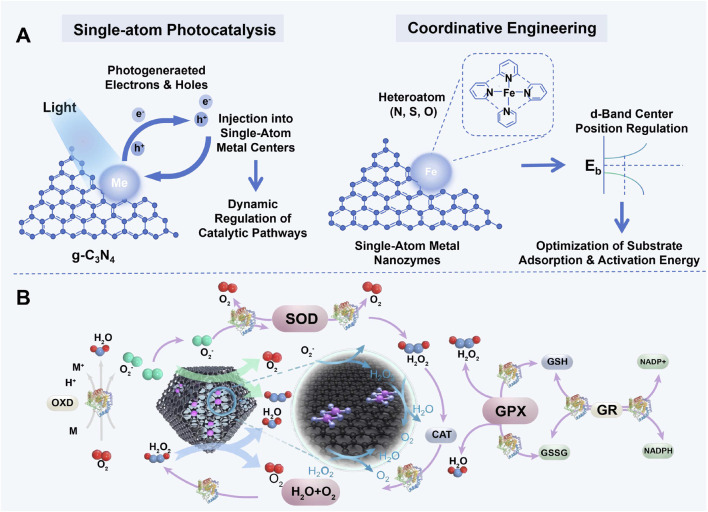
Schematic diagram of single-atom photocatalysis and coordination engineering strategies. **(A)** Multi-enzyme catalytic cascade of Fe_2_-NCs nanozymes. Reproduced from (Ying et al., 2026). Copyright 2026 Advanced Science **(B)**.

Single-atom catalysis, in which isolated metal atoms are anchored on specific supports, provides an atomically precise platform for designing high-performance photo-responsive nanozymes (Jiao et al., 2020). Compared with conventional nanoparticle catalysts, single-atom catalysts maximize metal-atom utilization and exhibit unique catalytic properties owing to their well-defined electronic structures and coordination environments (Chen et al., 2026). By precisely regulating the coordination environment around the metal center, for example, by introducing N, S, or O atoms as coordination ligands, the d-band center of the active site can be finely adjusted (Figure 4A). This modulation optimizes the adsorption energy and activation capability toward key reaction substrates (e.g., H_2_O_2_, O_2_) (Fu et al., 2020). Heteroatoms with different electronegativities and coordination capabilities can significantly influence the charge density and spin state of the metal center, thereby regulating the strength of orbital hybridization between the active site and substrate molecules.

Common supports, including graphitic carbon nitride (g-C_3_N_4_), metal-organic frameworks (MOFs), and carbon-based materials, can effectively stabilize isolated metal atoms and prevent their aggregation because of their abundant surface functional groups and adjustable electronic structures (Rocha et al., 2023). The substrate material fundamentally influences the electronic structure, catalytic mechanism, and overall performance of nanozyme systems through three primary interfacial coupling mechanisms. There are: (i) charge transfer modulation, where electronic coupling between substrate and catalytic center alters local electron density at active sites; (ii) defect engineering, where intrinsic or introduced defects serve as additional active sites or modulate the electronic structure of neighboring catalytic centers; and (iii) geometric confinement effects, where nanoporous or layered substrates confine active sites within well-defined nanospaces to enhance catalytic selectivity and turnover frequency (He W. et al., 2024). While carbon-based supports have been widely employed due to their excellent conductivity, recent advances demonstrate that non-carbon substrates offer distinct advantages for modulating charge transfer, interfacial electronic structures, and catalytic kinetics in nanozyme systems. For example, molybdenum disulfide (MoS_2_) exhibits intrinsic POD, oxidase (OXD), CAT, and superoxide dismutase (SOD)-like activities, enabling complex cascade reactions without relying on additional natural enzymes (Peng et al., 2026a). Beyond single-atom configurations, recent advances have extended coordination engineering to dual-atom nanozymes. For example, in the Fe-N-C system, the strong electron-donating ability of N atoms endows the Fe center with high electron density, which facilitates O-O bond cleavage in H_2_O_2_ and contributes to oxidative stress mitigation and delayed osteoarthritis progression (Figure 4B) (Ying et al., 2026). This dual-atom architecture more closely mimics the bimetallic active centers found in natural metalloenzymes, representing a promising direction for overcoming the intrinsic activity limitations of single-atom nanozymes.

Under irradiation with specific wavelengths of light, photosensitive supports (e.g., g-C_3_N_4_) or photo-responsive single-atom sites can generate photogenerated electrons and holes. These charge carriers can be transferred to isolated metal centers, altering their oxidation states and electronic occupancy and thereby dynamically modulating catalytic pathways and reaction kinetics (Sun et al., 2024). This photo-regulation mechanism presents several distinct advantages. Firstly, the wavelength and intensity of light can be precisely controlled, enabling reversible or graded modulation of catalytic activity. Secondly, photoexcitation is non-invasive and avoids the additional toxicity associated with chemical stimulants. Thirdly, the photo-response is extremely rapid, achieving catalytic responses on the millisecond timescale. Furthermore, light irradiation may transform single-atom sites from an inactive state into a highly active state or alter catalytic product selectivity, thereby realizing intelligent responsive catalysis (Yuan et al., 2026; Zhuang et al., 2024). For example, a copper/zinc dual single-atom nanozyme (Cu/Zn-C_6_N_6_) exhibits excellent SOD-like activity under dark conditions, but it can efficiently and reversibly switch to distinct POD-like activity upon light irradiation, thereby achieving photocontrolled switching of substrate selectivity (Hong et al., 2026). Moreover, in tumor therapy, photo-responsive single-atom nanozymes can be selectively activated at the tumor site using light of specific wavelengths, thereby maximizing therapeutic efficacy while reducing damage to surrounding healthy tissues (Shi X. et al., 2025). This photocontrol ability endows single-atom nanozymes with considerable potential for precision catalytic therapy.

## 3 Catalytic enhancement mechanisms in photo-responsive nanozymes

### 3.1 Catalytic enhancement driven by photo-generated carriers

**FIGURE 5:**
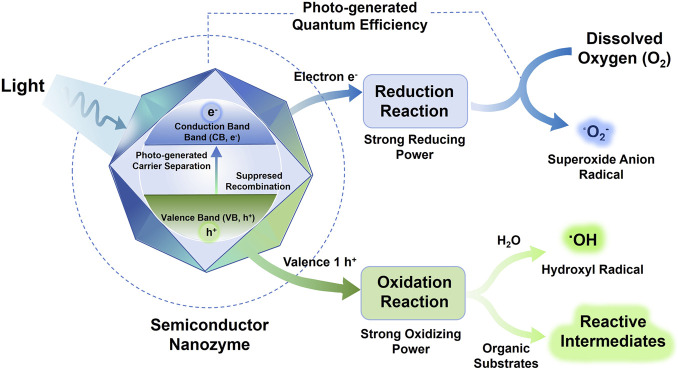
Mechanism diagram for catalytic activity via photogenerated charge carriers.

The catalytic enhancement of photo-responsive nanozymes is fundamentally associated with the redox activity of photogenerated electron-hole pairs generated at the surface of photoactive nanomaterials (Li et al., 2025a; Xu et al., 2026b). Upon irradiation with photons of appropriate energy, electrons in the valence band of semiconductor nanomaterials can be promoted to the conduction band, thereby generating conduction band electrons (e^−^) and valence band holes (h^+^). These high-energy charge carriers can directly participate in or modulate catalytic reactions on the nanomaterial surface. The reaction routes and catalytic efficiencies depend on the band-edge positions of the semiconductor, the density of surface active sites, and the adsorption behavior of reaction substrates (Liu Y. et al., 2025). Photogenerated electrons possess strong reductive capability and can reduce dissolved oxygen (O_2_) to superoxide anion radicals (·O_2_^−^), which are pivotal reactive intermediates in numerous photocatalytic oxidation processes (Li et al., 2026f). Conversely, photogenerated holes exhibit strong oxidative capability and can directly oxidize water molecules (H_2_O) or organic substrates adsorbed on the material surface, leading to the generation of hydroxyl radicals (·OH) or other reactive intermediates that drive oxidation reactions (Figure 5) (Li L. et al., 2020).

The efficient separation and utilization of photogenerated charge carriers are crucial determinants of the catalytic performance of photo-responsive nanozymes. Photogenerated electrons and holes readily recombine on a nanosecond to microsecond timescale, dissipating absorbed energy through non-radiative relaxation or fluorescence emission, thereby reducing the density of effective charge carriers available for surface catalytic reactions (Li K. et al., 2024; Liu et al., 2022). To suppress charge carrier recombination, various strategies have been developed, including heterojunctions construction, defect engineering, surface cocatalyst loading, and the application of external electric fields. For example, in TiO_2_/Au composite systems, Au nanoparticles can act not only as plasmonic light-harvesting enhancers but also as electron traps to capture photogenerated electrons, thereby extending charge carrier lifetimes (Kang et al., 2025). Furthermore, precise regulation of charge carrier behavior is crucial for tailoring nanozyme functions toward specific therapeutic applications. In tumor therapy, preferential generation of highly oxidative species (e.g., ·OH) is desirable for inducing tumor cell damage (Zhang T. et al., 2026). Conversely, balanced utilization of electrons and holes may be required to achieve broad-spectrum ROS generation for microbial eradication (Xu et al., 2026a). The relationship between charge carrier migration distance and material dimensions also deserves attention. When semiconductor particles are reduced to the nanoscale, particularly below 10 nm, the diffusion distance from the bulk to the surface is significantly reduced. This reduction decreases recombination probability and improves the quantum efficiency of surface catalytic reactions (Yin et al., 2024). Therefore, the rational design of nanozyme size, morphology, surface defects, and interfacial structures is essential for maximizing the utilization efficiency of photogenerated charge carriers.

### 3.2 Catalytic enhancement mechanism via plasmonic resonance effects

**FIGURE 6:**
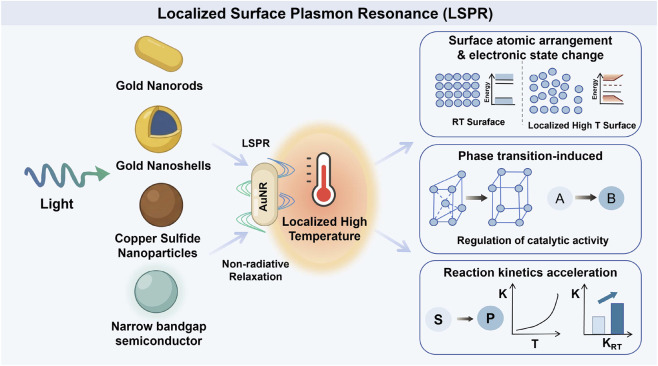
Mechanism diagram for catalytic activity via plasmonic resonance effects.

Plasmonic resonance effect represents another important mechanism for enhancing the catalytic efficiency of nanozyme. When the frequency of incident photons matches the collective oscillation frequency of free electrons in noble-metal nanostructures, LSPR is generated. Subsequently, the absorbed light energy is efficiently converted into heat through non-radiative relaxation processes, resulting in a localized temperature increase around the nanozyme surface (Wu Q. et al., 2025). This photothermal effect is particularly pronounced in noble metal nanostructures with strong localized surface plasmon resonance (e.g., gold nanorods, gold nanoshells) and narrow-bandgap semiconductors (e.g., copper sulfide, tungsten oxide) (Shao et al., 2023; Wang J. et al., 2025; Yang et al., 2023). From a thermodynamic perspective, the local temperature elevation induced by plasmonic resonance leads to an exponential increase in reaction rate constants with temperature. In addition, localized heating can alter surface atomic arrangement and electronic structure, and may even induce phase transitions, thereby modulating the catalytic activity of nanozyme materials (Figure 6) (Fu et al., 2025).

Under NIR irradiation, nanomaterials with high photothermal conversion efficiency can efficiently transform light energy into heat and generate localized high temperatures that accelerate catalytic reactions (Du et al., 2025). For example, porous nanoflowers assembled from three-dimensionally stacked molybdenum disulfide nanosheets exhibit enhanced photothermal conversion capability and peroxidase-like activity due to their abundant porous structures and large specific surface areas. Upon light irradiation, the photothermal effect synergistically enhances catalytic activity and promotes ROS generation, thereby enabling imaging-guided combination therapy (Jiang et al., 2020). Furthermore, a Fe-N-C flower-like nanoscale cascade reactor loaded with AuPt nanoparticles exhibits improved photothermal conversion capability through the introduction of AuPt nanoparticles. Under NIR irradiation, this system enables mild photothermal therapy (PTT) and utilizes the photothermal effect to accelerate multienzyme-like cascade catalytic reactions such as glucose oxidase-, catalase-, and peroxidase-like activities. This process establishes a self-sufficient H_2_O_2_/O_2_ cycling system within tumor cells, and significantly improves therapeutic efficacy against hypoxic tumors (Guo et al., 2025). These studies indicate that the plasmonic resonance-induced photothermal effect not only supplies localized heat but also creates more favorable thermodynamic and kinetic conditions for nanozyme-mediated catalytic reactions. Therefore, precise control over the plasmonic resonance intensity and the spatial distribution of plasmonic resonance-induced photothermal effects is critical for achieving efficient therapy with photo-responsive nanozymes.

### 3.3 Multi-mechanism synergistic catalytic strategies

Photogenerated charge carrier-mediated redox processes and plasmonic resonance effects represent the two major mechanisms for catalytic enhancement in photo-responsive nanozymes. However, a single enhancement mechanism is often insufficient to address the complexities of pathological environments, particularly with respect to catalytic efficiency, spatiotemporal controllability, and therapeutic precision (Zhang M. et al., 2026). In recent years, increasing attention has been directed toward multi-mechanism synergistic catalytic strategies, which improve therapeutic performance and overcome the intrinsic limitations of individual modalities (Li et al., 2025b).

A natural synergy exists between plasmonic resonance effects and photogenerated charge carrier-mediated photocatalysis (Xia et al., 2026). The localized surface plasmon resonance of noble metal nanoparticles can generate intense local electromagnetic field enhancement and hot electron injection effects. These high-energy hot electrons can be directly injected into the conduction band of adjacent semiconductors, thereby increasing the concentration of free electrons available for catalytic reactions (Wei et al., 2026). Simultaneously, plasmon-induced local electromagnetic fields can facilitate the polarization of molecules adsorbed on the semiconductor surface, thereby reducing the energy barrier of redox reactions (Zhong et al., 2025). For example, in dumbbell-like AuNR@CeO_2_ hybrid nanozymes, hot electrons transfer from AuNRs to CeO_2_ modulates the Ce^3+^/Ce^4+^ ratio, resulting in a significant enhancement of peroxidase-like activity (Yang et al., 2025).

Photothermal-photocatalytic synergy is mainly reflected in the acceleration of catalytic reaction kinetics through localized heating. Under NIR irradiation, nanomaterials with high photothermal conversion efficiency can convert light energy into heat and create localized high-temperature regions on the nanozyme surface, thereby reducing the activation energy of catalytic reactions (Liu et al., 2026; Zhang X. et al., 2026). Meanwhile, moderate temperature elevation can modulate catalytic pathways, alter the balance between substrate adsorption and desorption, and promote product release (Sun et al., 2025). For example, in the Fe_NC-Pd_NC system, the electron-withdrawing effect of Pd_NCs induces spin-state rearrangement of Fe_(II) from a low-spin to an intermediate-spin state. This transition promotes the heterolytic cleavage pathway of H_2_O_2_ and accelerates H_2_O desorption, thereby selectively enhancing peroxidase-like activity without increasing oxidase-like activity (Wei et al., 2023). Furthermore, photocatalytic effects can effectively overcome the oxygen-dependent limitations of conventional photodynamic therapy. Nanozymes with catalase-like activity can decompose endogenous H_2_O_2_ to generate O_2_ *in situ*, thereby alleviating hypoxia-mediated inhibition of photodynamic therapy (Wang K. et al., 2026). Therefore, multi-mechanism synergy is not merely an additive combination of individual mechanisms, but rather enables amplified catalytic performance through the coordinated integration of energy transfer, substrate supply, and catalytic pathway.

## 4 Applications of photo-responsive nanozymes in precision medicine

### 4.1 Applications of photo-responsive nanozymes in tumor therapy

**FIGURE 7:**
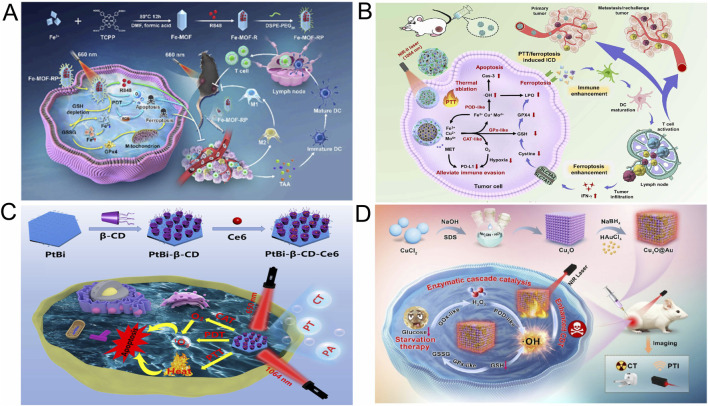
Applications of photo-responsive nanozymes in tumor therapy. **(A)** Schematic illustration of synthesis and function of Fe-TCPP MOFs. Reproduced from (Fan et al., 2024). Copyright 2024 ACS Nano. **(B)** Schematic illustration of MCMSFT for cancer treatment. Reproduced from (Zhang et al., 2025a). Copyright 2025 Science Advances. **(C)** Schematic illustrations for the construction of PtBi‐β-CD-Ce6 Nanozymes. Reproduced from (Xie et al., 2022b). Copyright 2022 Inorganic Chemistry. **(D)** Schematic diagram for the preparation of the Cu_2_O@Au nanozyme and antitumor mechanism. Reproduced from (Li et al., 2024a). Copyright 2024 ACS Nano.

Photo-responsive nanozymes integrate catalytic therapy, PDT, and PTT, enabling synergistic anti-tumor effects that exceed those of individual monotherapies. This synergy is primarily based on the ability of nanozymes to respond to the tumor microenvironment (TME) while using light energy to activate multiple cytotoxic mechanisms simultaneously (Zheng et al., 2026). The TME is characterized by conditions such as low pH, high H_2_O_2_ level, hypoxia, and glutathione (GSH) overexpression (Guo W. et al., 2026). These features not only facilitate malignant tumor progression but also provide endogenous stimuli for the rational design of TME-responsive nanozymes. For example, photo-responsive nanozymes can specifically remodel the hypoxic and immunosuppressive tumor microenvironment. These systems not only catalyze the decomposition of hydrogen peroxide to generate oxygen, thereby enhancing photodynamic therapy, but also induce ferroptosis in tumor cells by depleting glutathione and releasing iron ions (Figure 7A) (Fan et al., 2024). Under dual stimulation by the tumor microenvironment and 1,064 nm laser irradiation, the intelligent nanozyme system MCMSFT synergistically induces apoptosis, ferroptosis, and necroptosis, while activating antitumor immune responses through hypoxia alleviation, GSH depletion, PD-L1 downregulation, and related mechanisms (Figure 7B) (Zhang H. et al., 2025). Furthermore, nanozymes with catalase-like activity can decompose overexpressed hydrogen peroxide in the TME to generate oxygen *in situ*. This process effectively alleviates tumor hypoxia and significantly improves the ability of photosensitizers to generate singlet oxygen (^1^O_2_) upon light irradiation, thereby enhancing the efficacy of PDT (Figure 7C) (Wang et al., 2020; Xie Y. et al., 2022). Additionally, some nanozymes, such as Cu_2_O@Au, can disrupt tumor energy homeostasis, thereby inhibiting tumor growth and enabling synergistic starvation therapy, chemodynamic therapy, and PTT (Figure 7D) (Li et al., 2024c). In summary, photo-responsive nanozymes are not only direct tumor-killing tools but also potent modulators of the immune system. These systems can efficiently eradicate tumor cells and activate anti-tumor immune responses through the induction of immunogenic cell death. This approach effectively overcomes the oxygen dependency of conventional PDT and the potential thermo-resistance associated with PTT alone, thereby providing a more powerful and precise strategy for tumor therapy.

### 4.2 Applications of photo-responsive nanozymes in microbial eradication

**FIGURE 8:**
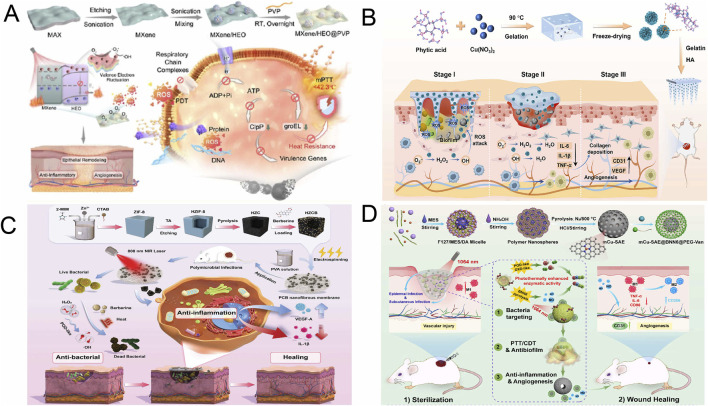
Applications of photo-responsive nanozymes in microbial eradication. **(A)** Schematic of the MHP system achieving PDT/mPTT multimodal antibacterial effects. Reproduced from (Zhang et al., 2025b). Copyright 2025 ACS Nano. **(B)** Schematic illustration for the preparation and application of Cu-MOG MN patch in promoting bacteria-infected wound healing. Reproduced from (He et al., 2026). Copyright 2026 Advanced Healthcare Materials. **(C)** The fabrication process of PCB nanofibrous membranes and their mechanism of bactericidal action. Reproduced from (Dong et al., 2024). Copyright 2024 Advanced Healthcare Materials. **(D)** A schematic diagram illustrating the treatment of diabetic epidermal wounds and subcutaneous cysts using CBPV. Reproduced from (Chen et al., 2025a). Copyright 2025 Advanced Science.

Photo-responsive nanozymes represent an emerging strategy for microbial eradication. Their primary antibacterial mechanism involves the utilization of light energy to activate nanozymes, resulting in the production of high concentrations of ROS. This process can efficiently disrupt bacterial biofilms and cell membranes/walls, thereby killing drug-resistant bacteria with a relatively low risk of inducing bacterial resistance (Wang X. et al., 2021). Unlike conventional antibiotics that typically inhibit specific bacterial metabolic enzymes or cell wall synthesis, ROS can simultaneously attack multiple biological targets, including membrane lipids, proteins, and nucleic acids. This multi-target mode of action makes it difficult for bacteria to develop resistance through single-point mutations (Figure 8A) (Ahmad et al., 2025; Generalova and Dushina, 2025; Zhang et al., 2025c). For example, chitosan-stabilized selenium nanoparticles (CS@Se_NPs) exhibit light-stimulated oxidase-like activity under visible blue light irradiation at 420 nm, enabling complete eradication of bacteria and biofilms within 5–15 min. Their antibacterial efficacy is achieved through synergistic mechanisms, including ROS generation, membrane destabilization, photocatalytic inactivation, and ferroptosis-like bacterial death (Zhao et al., 2025). This ROS-mediated attack directly compromises critical bacterial structures, thereby circumventing the target specificity of conventional antibiotics and reducing the risk of bacterial resistance (Zhu et al., 2022).

Based on the specific characteristics of bacterial infection microenvironment, intelligent responsive nanozymes have been developed for microbial eradication (He et al., 2025; Wen et al., 2025). For instance, a pH-responsive microneedle system encapsulating multifunctional metal-organic gel nanozymes (Cu-MOG MN) can intelligently switch its catalytic functions in response to pH changes during different stages of infected wound healing. In the acidic infectious microenvironment, the Cu-MOG MN system activates a SOD-POD cascade, triggering a burst of ROS to efficiently eradicate drug-resistant bacteria and disrupt biofilms. Once the wound microenvironment returns to a neutral pH, its enzymatic activity switches to dual superoxide dismutase-glutathione peroxidase activity, enabling precise scavenging of excessive ROS and preventing tissue damage (Figure 8B) (He et al., 2026). This microenvironment-responsive design enables selective nanozyme activation at infected sites, thereby improving therapeutic specificity and efficacy.

NIR activation provides a promising route for the non-invasive treatment of deep-seated tissue infections (Figure 8C) (Dong et al., 2024; Song et al., 2025). Compared with conventional NIR-I light at 700–950 nm, NIR-II light offers deeper tissue penetration and a higher maximum permissible exposure. These characteristics make it more suitable for treating infections located in deeper tissues (Chen P. et al., 2025; Ding et al., 2025; Li N. et al., 2026; Zhou et al., 2024; Zhu X. et al., 2026). For example, the multifunctional therapeutic nanoplatform mCu-SAE@BNN6@PEG-Van (CBPV) integrates bacteria-targeting capability with an NIR-II-responsive therapeutic cascade. Under 1,064 nm laser irradiation, CBPV enables deep-tissue photothermal activation, thermally enhanced chemodynamic activity, and controlled nitric oxide (NO) release (Figure 8D) (Chen J. et al., 2025). Collectively, these studies indicate that combining photo-responsive catalytic activity with light-induced physical effects is a crucial direction for the development of high-efficiency and low-resistance antimicrobial nanozyme platforms.

### 4.3 Applications of photo-responsive nanozymes in inflammatory disease therapy

The pathogenesis of inflammatory diseases is closely associated with oxidative stress imbalance, and excessive ROS production is a major contributor to tissue damage and inflammatory cascades amplification (Li et al., 2026j). Under normal physiological conditions, ROS act as signaling molecules involved in immune regulation and tissue repair (Li et al., 2024b). However, when ROS generation exceeds the scavenging capacity of endogenous antioxidant system, oxidative damage occurs, including lipid peroxidation, protein carbonylation, and DNA damage. These processes can activates the nuclear factor kappa B (NF-κB) signaling pathway and promote the expression of pro-inflammatory cytokines, such as interleukin-1β (IL-1β) and tumor necrosis factor-α (TNF-α), thereby establishing a positive feedback loop between oxidative stress and inflammation (Li et al., 2026c; Zhang Y. et al., 2025). Therefore, developing photo-responsive nanozymes capable of precisely scavenging excessive ROS is crucial for alleviating oxidative stress and controlling inflammation progression.

**FIGURE 9:**
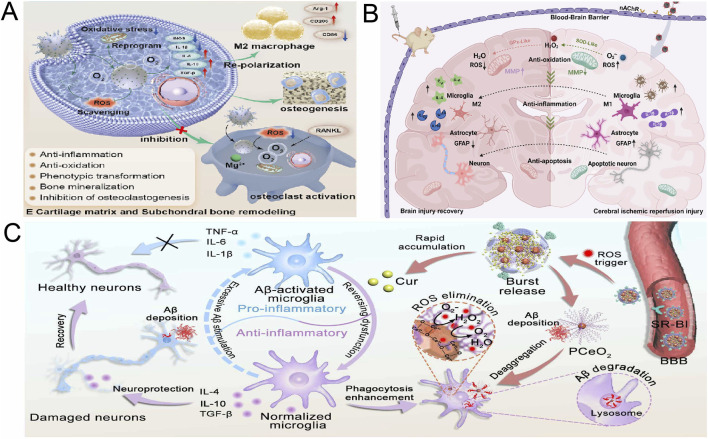
Applications of photo-responsive nanozymes in inflammatory disease therapy. **(A)** A schematic diagram of the therapeutic mechanism of CPMP nanozyme for OA (Shi X. et al., 2025). Copyright 2025 Advanced Functional Materials. **(B)** Schematic illustration of the therapeutic mechanism of T-mPDA-Cu/Se for cerebral ischemia reperfusion injury. Reproduced from (Wu et al., 2025c). Copyright 2025 Advanced Functional Materials. **(C)** Illustration of APPCeO_2_/Cur preparation for reversing microglial dysfunction. Reproduced from (Han et al., 2025). Copyright 2025 Science Advances.

Recently, photo-responsive nanozymes with SOD-like and CAT-like activities have attracted increasing attention because they combine the intrinsic advantages of conventional nanozymes with spatiotemporally regulated catalytic activity (Cai et al., 2026). SOD-like activity catalyzes the dismutation of superoxide anions (·O_2_^−^) into H_2_O_2_ and O_2_, whereas CAT-like activity further decomposes H_2_O_2_ into H_2_O and O_2_. This cascade-like antioxidant mechanism enables efficient scavenging of pathogenic ROS (Singh et al., 2023; Yan M. et al., 2025; Zhu K. et al., 2026). In arthritis therapy, combining the antioxidant capacity of CeO_2_ nanozymes with the photothermal responsiveness of polydopamine effectively addresses the challenge of precise delivery of active ingredients in osteoarthritis treatment, thereby achieving synergistic cartilage protection and subchondral bone regeneration (Figure 9A) (Shi G. et al., 2025). CeO_2_ exhibits excellent ROS scavenging capability owing to its mixed Ce^3+^/Ce^4+^ valence states and oxygen vacancies. The cyclic catalytic mechanism underlying its SOD-like and CAT-like activities enables efficient ROS scavenging at low concentrations without rapid inactivation. In periodontitis therapy, Ce6@PEG-MoO_X_ hydrogels significantly inhibit alveolar bone resorption and reduce gingival inflammatory infiltration. The released MoO_X_ nanozymes alleviate cellular oxidative stress and generate O_2_ *in situ*, thereby enhancing Ce6-mediated PDT for deep biofilm eradication. After bacterial eradication, residual ROS can be further scavenged, and finally the precise regulation of ROS is realized (Qin et al., 2026).

Furthermore, biomimetic cascade nanozymes have been constructed to actively cross the blood-brain barrier for the treatment of cerebral ischemia-reperfusion injury. These nanozymes efficiently scavenge ROS during reperfusion injury by mimicking the cascade activities of SOD and GPx, thereby alleviating neuroinflammation and inhibiting neuronal apoptosis (Figure 9B) (Wu et al., 2025b). In neuroinflammation therapy, photo-responsive nanozymes also demonstrate distinct advantages. Studies have shown that CeO_2_ nanozymes with SOD-like and CAT-like activities can effectively scavenge excessive ROS generated around β-amyloid aggregates, thereby reducing microglia-mediated neuroinflammatory responses (Figure 9C) (Han et al., 2025). In conclusion, photo-responsive nanozymes enable intelligent intervention in inflammatory microenvironments by regulating ROS generation and scavenging. This approach provides a promising strategy for the precision therapy of inflammatory diseases, including arthritis, periodontitis, and neuroinflammation.

### 4.4 Applications of photo-responsive nanozymes in biosensing

**FIGURE 10:**
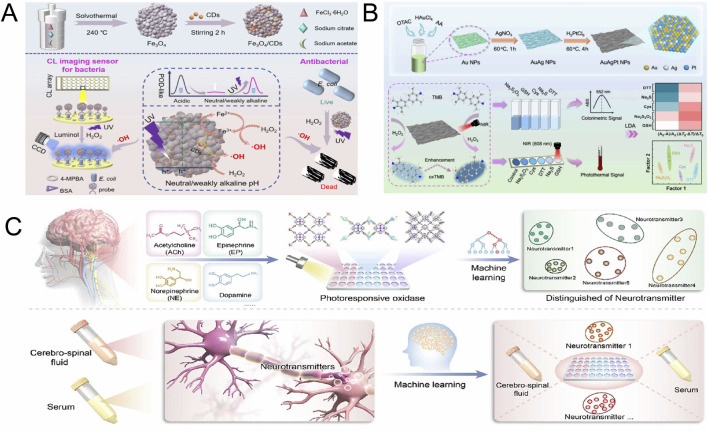
Applications of photo-responsive nanozymes in biosensing. **(A)** Schematic for preparing Fe3O4/CDs and the strategy for a CL imaging sensor to detect and eliminate bacteria. Reproduced from (Peng et al., 2025a). Copyright 2025 Analytical Chemistry. **(B)** Schematic illustration of the colorimetric/photothermal dual-signal sensing array based on AuAgPt NPs for discriminating SCCs. Reproduced from (Wu et al., 2026a). Copyright 2026 Analytical Chemistry. **(C)** Schematic illustration of Zn_TCPP MOF-based light-responsive nanozyme sensor array for neurological disease diagnosis. Reproduced from (Yu et al., 2025). Copyright 2025 Advanced Science.

Biosensing plays a crucial role in early disease diagnosis, prognosis evaluation, and therapeutic monitoring through the sensitive and selective detection of biomarkers (Su et al., 2022; Wang M. et al., 2023; Wang X. et al., 2025). The advantages of photo-responsive nanozymes in biosensing are primarily reflected in three aspects. Firstly, light serves as an external stimulus that enables spatiotemporally controlled activation of sensing signals, thereby reducing background interference. Secondly, the catalytic signal amplification capability of nanozymes can convert weak biorecognition events into detectable optical, electrical, or thermal signals. Thirdly, the multimodal properties of photo-responsive nanozymes facilitate self-calibrated or ratiometric sensing strategies, thereby improving detection accuracy (Liu Y. et al., 2020; Panferov and Liu, 2024). For example, a chemiluminescence imaging immunosensor has been developed that achieves highly sensitive and selective bacterial detection while also demonstrating potent *in vitro* antibacterial activity (Figure 10A) (Peng et al., 2025b). Additionally, a near-infrared-responsive AuAgPt trimetallic plasmonic nanozyme was developed to construct a dual-signal sensing array based on plasmon-enhanced peroxidase-like activity and photothermal performance. By integrating linear discriminant analysis, this sensing platform enabled discrimination, identification, and quantitative detection of five sulfur-containing compounds (Figure 10B) (Wu et al., 2026a). Furthermore, a ternary sensor array based on a Zn_TCPP MOF photo-responsive nanozymes was proposed for multiplex neurotransmitter detection. This array utilizes light-controlled catalytic reactions to improve detection accuracy, thereby enabling rapid, sensitive, and multiplexed neurotransmitter analysis. This method provides potential value for early diagnosis and continuous monitoring of neurological diseases (Figure 10C) (Yu et al., 2025). Overall, the application of photo-responsive nanozymes in biosensing is expanding from *in vitro* diagnostics towards *in vivo* dynamic monitoring. Their light-controllable catalytic activity, efficient signal amplification capability, and multimodal sensing characteristics provide a foundation for developing highly sensitive and selective sensing platforms.

## 5 Challenges and future perspectives

### 5.1 Biocompatibility and long-term toxicity

Photo-responsive nanozymes hold promise for biomedical applications, but their clinical translation still faces several challenges. Among them, biocompatibility and long-term toxicity are primary issues that require systematic evaluation (Guo M. et al., 2026). Especially for nanozymes containing noble metals or rare-earth elements, their biocompatibility, metabolic pathways, and potential long-term toxicity require further investigation (Zeng et al., 2026). Although metal-free nanozymes may avoid certain toxicity and stability issues associated with metal-based systems, their long-term biological effects still require comprehensive assessment (Choi et al., 2026). The ideal photo-responsive nanozymes should be degraded into non-toxic metabolites after completing their therapeutic functions and subsequently be cleared through the kidney or liver pathways. Recent studies have demonstrated that Cu/Mn NC dual-atom nanozymes can be degraded in the tumor microenvironment. The released Cu^2+^ and Mn^2+^ can be excreted through metabolic pathways, and the pathological microenvironment serves as a degradation trigger, thereby achieving spatiotemporal separation between therapeutic function and degradation clearance (Li Z. et al., 2025). Therefore, future studies should establish standardized biocompatibility evaluation systems for photo-responsive nanozymes, and develop high-sensitivity *in vivo* tracing technologies to precisely monitor the biodistribution, metabolic transformation and clearance kinetics of nanozymes.

### 5.2 Catalytic efficiency and selectivity

The catalytic efficiency, selectivity, and stability of photo-responsive nanozymes in complex physiological environments remain major barriers. In biological media, the surface of nanozymes can rapidly adsorb proteins and form protein coronas. These coronas may shield catalytically active sites and alter surface charge and hydrophobicity, resulting in a significant decrease in catalytic efficiency (Ding et al., 2026). Furthermore, dynamic variations in pH, ionic strength and biomolecules within physiological environments can disturb the catalytic activity of nanozymes and may induce non-specific catalytic damage to healthy tissues (Feng et al., 2022). Therefore, improving catalytic performance, substrate selectivity and long-term stability under complex biological environments is a central challenge for achieving precise therapeutic applications. Researchers have found that endowing nanozymes with multiple enzyme-like activities has emerged as an effective strategy to enhance their adaptability to the complex and variable pathological microenvironments (Chen et al., 2024; Chen W. et al., 2025; He S. et al., 2024; Li et al., 2026e). For example, the nanozyme PEG/Ce-Bi@DMSN with a bacteria-mimicking morphology possesses photothermal-enhanced dual enzyme-mimicking activities and GSH depletion function. In the acidic tumor microenvironment, this nanozyme displays both POD-like and CAT-like activities and generates OH and oxygen to alleviate hypoxia. Additionally, it consumes GSH through redox reactions and weakens the antioxidant defense capability of tumor cells. The catalytic performance is further improved by the photothermal effect of Bi_2_S_3_ (Dong et al., 2020). Therefore, photo-responsive nanozymes can more intelligently respond to pathological signals, perform complex catalytic cascades, and ultimately achieve efficient and precise disease therapy through multifunctional design.

### 5.3 Penetration depth and tissue specificity

The contradiction between light penetration depth and tissue-specific activation remains the main limitation of photo-responsive nanozymes for the treatment of deep-seated diseases (Pan B. et al., 2026; Qu et al., 2025). At present, many efficient photocatalytic processes still rely on ultraviolet or visible light excitation, but these wavelengths exhibit limited penetration in biological tissues (Li B. et al., 2026; Zhang L. et al., 2025). Therefore, the development of photoresponsive nanozymes that can be activated by NIR or longer-wavelength light is of great importance for deep-tissue biomedical applications (Feng et al., 2026). NIR-II (1,000–1700 nm) can achieve light-controlled therapy for deep-seated lesions owing to its reduced tissue scattering, lower absorption and improved penetration depth (Li W. et al., 2020). Furthermore, the advancement of NIR-II-responsive nanozymes with narrow-bandgap (<1.2 eV) represents an important direction. For example, Co-O-Mn bridged dual-atom nanozymes responsive to 1,550 nm irradiation demonstrate the feasibility of long-wavelength photoactivation. Through rational band-structure engineering, these materials can promote the generation and separation of photogenerated charge carriers and reduce the risk of tissue damage caused by excessive thermal effects (Li C. et al., 2026). Therefore, the realization of deep-tissue photoactivation through band engineering (e.g., the construction of a narrow-bandgap semiconductors or the incorporation of upconversion materials) represents an important technological pathway to advance the clinical transformation of photo-responsive nanozymes.

In summary, photo-responsive nanozymes represent an emerging field of photocatalytic technology and nanozyme catalysis. With the maturation of photocatalytic theory and advances in nanofabrication technologies, rapid progress has been made in the rational design of light-controlled nanozyme systems. Through strategies such as plasmonic resonance, heterojunction engineering, and single-atom catalysis, these intelligent nanozymes have demonstrated great potential in photodynamic and photothermal synergistic tumor therapy, the eradication of drug-resistant bacterial infections, and regulation of the inflammatory microenvironment. These advances indicate that nanozyme research is moving beyond conventional enzyme-mimetic catalysis toward spatiotemporally controllable precision intervention. In the future, research on the core challenges of biocompatibility, catalytic efficiency, and light penetration depth will realize their value in precision medicine.
